# Incidence, determinants and prognostic relevance of dyspnea at admission in patients with Takotsubo syndrome: results from the international multicenter GEIST registry

**DOI:** 10.1038/s41598-020-70445-9

**Published:** 2020-08-12

**Authors:** Luca Arcari, Maria Beatrice Musumeci, Thomas Stiermaier, Ibrahim El-Battrawy, Christian Möller, Federico Guerra, Giuseppina Novo, Enrica Mariano, Luca Rosario Limite, Luca Cacciotti, Raffaella Semeraro, Massimo Volpe, Francesco Romeo, Pasquale Caldarola, Holger Thiele, Ibrahim Akin, Natale Daniele Brunetti, Ingo Eitel, Francesco Santoro

**Affiliations:** 1 Cardiology Department, Clinical and Molecular Medicine Department, Faculty of Medicine and Psychology, Sapienza University of Rome, Rome, Italy; 2 Medical Clinic II (Cardiology/Angiology/Intensive Care Medicine) and German Center for Cardiovascular Research (DZHK), University Heart Center Lübeck, Partner Site Hamburg/Kiel/Lübeck, Lübeck, Germany; 3 First Department of Medicine, Faculty of Medicine, University Medical Centre Mannheim (UMM) University of Heidelberg, Mannheim, Germany and German Center for Cardiovascular Research, Partner Site, Heidelberg-Mannheim, Mannheim, Germany; 4 Cardiology and Arrhythmology Clinic, Marche Polytechnic University, University Hospital “Umberto I – Lancisi – Salesi”, Ancona, Italy; 5 Cardiology Unit, Biomedical Department of Internal Medicine and Medical Specialties, University of Palermo, Palermo, Italy; 6 Division of Cardiology, University of Rome Tor Vergata, Rome, Italy; 7 Cardiology Unit, Madre Giuseppina Vannini Hospital, Rome, Italy; 8 IRCCS Neuromed, Pozzilli, Italy; 9 San Paolo Hospital, Bari, Italy; 10 Department of Internal Medicine/Cardiology and Leipzig Heart Institute, Heart Center Leipzig at University of Leipzig, Leipzig, Germany; 11 Department of Medical and Surgical Sciences, University of Foggia, Viale Pinto n.1, 71122 Foggia, Italy

**Keywords:** Cardiology, Signs and symptoms

## Abstract

Clinical presentation of Takotsubo syndrome (TTS) may range from acute chest pain to dyspnea: the prognostic role of clinical onset is still controversial. Aim of this study was therefore to investigate the prognostic relevance of dyspnea at presentation in patients with TTS. We analyzed 1,071 TTS patients (median age 72 years, 90% female) enrolled in the international multicenter GEIST registry. Patients were divided according to the presence or absence of dyspnea at hospital admission, as clinically assessed by the accepting physician. The primary endpoint was occurrence of in-hospital complications defined as a composite of pulmonary edema, cardiogenic shock and death. Overall, 316 (30%) patients presented with dyspnea at hospital admission. Diabetes, lower left ventricular ejection fraction and presence of pulmonary disease or atrial fibrillation were independently associated with dyspnea. In-hospital pulmonary edema, cardiogenic shock and death (17% vs. 3%, p < 0.001; 12% vs. 7%, p = 0.009; 5% vs. 2%, p = 0.004 respectively) and long-term overall mortality (22% vs. 11%, p < 0.001) occurred more frequently in patients with dyspnea than in those without. At multivariable analysis, dyspnea at presentation remained independently associated to both the composite primary endpoint [odds ratio 2.98 (95% confidence interval (CI) 1.95–4.59, p < 0.001] and all-cause mortality [hazard ratio 2.03 (95% CI 1.37–2.99), p < 0.001]. Dyspnea at presentation is common in TTS and is independently associated with in-hospital complications and impaired long-term prognosis. Thorough symptom assessment including dyspnea therefore represents a valuable tool to potentially optimize risk-stratification models for TTS patients.

## Introduction

Takotsubo syndrome (TTS) is an acute heart failure syndrome characterized by transient left ventricular (LV) systolic dysfunction in the absence of identifiable culprit coronary artery stenosis^1^. Due to the reversible nature of LV dysfunction, it has been initially considered a disorder with a benign prognosis^2,3^. Nonetheless, several complications during in-hospital course and follow up may occur in TTS with an event rate comparable to that observed in patients with acute coronary syndrome (ACS)^4–7^. However, both short^8,9^ and long-term^6,10^ prognosis remain quite heterogeneous in TTS, which supports the need of improved risk stratification in order to identify those patients who may most benefit from intensive in-hospital management and long-term clinical follow-up^11^. Dyspnea at presentation is associated with worse prognosis in ACS^12^, and its assessment was able to improve accuracy of a traditional risk stratification model in these patients^13^. Thus, aim of the present study was to investigate the prevalence and prognostic relevance of dyspnea at admission in a large cohort of patients with TTS.

## Methods

Out of 1,303 patients enrolled in the multicentric international TTS registry GEIST (German Italian STress Cardiomyopathy registry), the final analysis included 1,071 patients (median age 72 years, 90% female) for which the data about dyspnea was available. Detailed description of inclusion and exclusion criteria have been previously reported^14,15^. Demographic, clinical and instrumental characteristics were recorded at admission. Symptoms at presentation, including the occurrence of dyspnea as a self-reported uncomfortable feeling of breathing, were recorded at admission by the accepting physician^13^. Ballooning patterns were described as apical (typical), mid-ventricular or basal as previously reported^16^. Recovery of left ventricular systolic function was documented 3–6 months after the acute event in all surviving patients. The primary end-point (in-hospital complications) was defined as the composite of acute pulmonary edema, cardiogenic shock needing supportive therapy (mechanical ventilator support, intra-aortic balloon pump, catecholamine administration), and in-hospital death. The secondary end-point was defined as all-cause long-term mortality. All events were verified via medical records and evaluated by a clinical events committee. All patients were managed in accordance with the Declaration of Helsinki and signed an informed consent for the processing of personal data for scientific research purpose. This is an observational study not needing full Ethics Committee approval. Data are not publicly available but would be available on request. Permission was obtained from the authorities to use the data.

### Statistical analysis

Data were analyzed with SPSS software version 22.0 (SPSS Inc., Chicago, Illinois). Continuous variables were presented as mean ± standard deviation or as median with interquartile range (25°–75°), as appropriate. Categorical variables were compared using a Chi-square analysis or Fisher’s exact test as appropriate. Normally distributed continuous variables were compared using the Student t test for independent samples, in case of non-normally distributed variables, Mann–Whitney U test was used. Univariate logistic regression analysis was used to calculate estimated and 95% confidence intervals odds ratios for variables associated to dyspnea and to in-hospital complications. Univariate Cox-regression analysis was performed to assess variables independently associated to long-term mortality. Variables with p < 0.1 on univariate analysis were entered into a multivariable logistic regression model to identify independent risk factors for the secondary end-point long-term mortality. Kaplan–Meier curve and log-rank test were used to assess survival function at follow-up. Landmark analysis at 30 days was performed to specifically assess long-term mortality excluding deaths occurred during the acute phase.

## Results

The incidence of dyspnea at admission was 30% (316 pts). Baseline demographic, clinical and instrumental characteristics of the whole study population and according to the presence or absence of dyspnea at presentation are reported in Table 1. Patients with dyspnea were older (78 vs. 74 years, p = 0.001), more frequently male (13% vs. 8%, p = 0.035), and less often presenting with chest pain (40% vs. 63%, p < 0.001). Among patients with dyspnea, a physical trigger was more often identified (38% vs. 31%, p = 0.03) and prevalence of comorbidities such as diabetes, pulmonary disease, and atrial fibrillation was higher (26% vs. 16%, p < 0.001; 38% vs. 13%, p < 0.001; 17% vs. 9%, p = 0.001 respectively) while LV ejection fraction (LVEF) was lower (36% vs. 40%, p < 0.001) compared to patients without dyspnea. Prevalence of dyspnea within specific physical trigger subgroups has been reported in supplementary Table 1.

**Table 1:** Baseline characteristics of study population.

Variable	Overall (n = 1,071)	Dyspnea (n = 316)	No dyspnea (n = 755)	p value
Age (years)	72 (63, 79)	78 (65, 80)	74 (63, 78)	**0.001**
Male	104 (10%)	40 (13%)	64 (8%)	**0.035**
**Coronary risk factors**				
Hypertension	725 (68%)	216 (69%)	509 (68%)	0.756
Diabetes mellitus	200 (19%)	82 (26%)	118 (16%)	** < 0.001**
Dyslipidemia	455 (42%)	129 (41%)	326 (43%)	0.490
Current smoker	230 (22%)	74 (24%)	156 (21%)	0.286
**Clinical presentation**				
Chest pain	603 (56%)	128 (40%)	475 (63%)	** < 0.001**
Hystory of cancer	141 (13%)	51 (16%)	90 (12%)	0.073
Pulmonary disease	215 (20%)	122 (38%)	93 (13%)	** < 0.001**
Stressful trigger^a^				
Emotional	435 (41%)	112 (35%)	323 (43%)	**0.026**
Physical	355 (33%)	120 (38%)	235 (31%)	**0.030**
None	285 (27%)	83 (26%)	202 (27%)	0.869
**Admission electrocardiographic findings**				
Atrial fibrillation	124 (12%)	54 (17%)	70 (9%)	**0.001**
ST-segment elevation	499 (47%)	132 (42%)	367(49%)	0.052
ST-segment depression	71 (7%)	26 (8%)	45 (6%)	0.240
T-wave inversion	501 (47%)	136 (56%)	365 (43%)	** < 0.001**
**Laboratory data**				
NT-Pro-BNP (161/1,071)^b^	7,561 (2,696, 15,500)	8,321 (3,431, 17,150)	7,511 (2,120, 15,337)	0.270
TnI peak (ng/ml) (433/1,071)^b^	2.9 (1.1, 6.3)	2.3 (0.68 – 5.1)	3.2 (1.38 – 6.8)	**0.013**
**Admission echocardiographic findings**				
Apical ballooning	942 (88%)	279 (88%)	663 (86%)	0.827
Mid-ventricular ballooning	111 (10%)	34 (11%)	77 (10%)	0.784
Basal ballooning	18 (2%)	3 (1%)	15 (2%)	0.228
Mitral Insufficiency (moderate to severe) (671/1,071)^b^	112 (17%)	50 (23%)	62 (14%)	**0.002**
EF (%)	40 (32, 45)	36 (30, 45)	40 (34, 45)	** < 0.001**
**Angiographic findings**				
CAD (721/1,071)^b^	114 (16%)	25 (13%)	89 (17%)	0.192
**Discharge therapy**				
Aspirin (774/1,071)^b^	551 (71%)	153 (62%)	398 (75%)	** < 0.001**
DAPT (336/1,071)^b^	43 (13%)	10 (8%)	33 (16%)	**0.035**
Anticoagulant (589/1,071)^b^	113 (19%)	60 (28%)	53 (14%)	** < 0.001**
Beta-Blocker (669/1,071)^b^	559 (84%)	177 (82%)	382 (84%)	0.336
Ace-inhibitor/ARBs (778/1,071)^b^	622 (80%)	198 (79%)	424 (80%)	0.720

Data are presented as no. (%), mean ± standard deviation, median (interquartile range).

Bold values are statistically significant.

*NT-pro-BNP* N-terminal prohormone of brain natriuretic peptide, *TnI* troponin I, *EF* left ventricular ejection fraction, *CAD* coronary artery disease, *DAPT* dual antiplatelet therapy, *ARBs* angiotensin II receptor blockers.

^a^Patients n = 22 experienced both emotional and physical trigger.

^b^Number of patients with available data.

At adjusted multivariable regression analysis, diabetes [OR 1.69 (95% confidence interval (CI) 1.15–2.48); p = 0.007], presence of pulmonary diseases [OR 4.1 (95% CI 2.83–5.9); p < 0.001], lower LVEF (per 10% decrease) [OR 1.26 (95% CI 1.06–1.5); p = 0.009] and presence of atrial fibrillation [OR 2.17 (95% CI 1.35–3.49); p = 0.001] were identified as factors independently associated with the occurrence of dyspnea (Table 2).

**Table 2:** Univariate and multivariable analysis for factors associated to dyspnea.

Variable	Univariate		Multivariable	
	OR (95% CI)	p	OR (95% CI)	p
Age (per 10 year)	1.23 (1.09–1.38)	** < 0.001**	NS	NS
Male	1.56 (1.03–2.38)	**0.036**	NS	NS
Physical trigger	1.35 (1.03–1.78)	**0.03**	NS	NS
History of cancer	1.41 (0.97–2)	0.074	NS	NS
Diabetes	1.9 (1.38–2.62)	** < 0.001**	1.69 (1.15–2.48)	**0.007**
Pulmonary disease	4.35 (3.11–6.1)	** < 0.001**	4.1 (2.83–5.9)	** < 0.001**
EF (per 10% decrease)	1.32 (1.14–1.51)	** < 0.001**	1.26 (1.06–1.5)	**0.009**
Atrial fibrillation	1.99 (1.33–2.98)	**0.001**	2.17 (1.35–3.49)	**0.001**
Apical ballooning	1.11 (0.75–1.65)	0.596	–	–

Bold values are statistically significant.

*OR* odds ratio, *CI* confidence interval, *EF* left ventricular ejection fraction, *NS* non-significant.

Overall, 167 (16%) patients experienced complications during the in-hospital course (Table 3). Patients with dyspnea at admission had higher in-hospital complications rates (29% vs. 10%; p < 0.001) compared with those with other or no symptoms at presentation; specifically, during hospital stay they suffered more frequently of both pulmonary edema and cardiogenic shock (17% vs. 3%, p < 0.001 and 12% vs. 7%, p = 0.009 respectively); moreover, median length of hospitalization was longer and in-hospital mortality higher (8 vs. 6 days, p < 0.001 and 5% vs. 2%, p = 0.004 respectively). After adjustment for other clinical risk factors in multivariable analysis, dyspnea was independently associated with the primary end-point in-hospital complications [OR 3 (95% CI 1.96–4.62); p < 0.001], along with male gender, lower LVEF, presence of pulmonary diseases, and atrial fibrillation (Table 4).

**Table 3:** In-hospital course and short- and long-term outcome.

Variable	Overall (n = 1,071)	Dyspnea (n = 316)	No dyspnea (n = 755)	p value
**In-hospital course**				
In-hospital complications	167 (16%)	90 (29%)	77 (10%)	** < 0.001**
Pulmonary edema	77 (7%)	54 (17%)	23 (3%)	** < 0.001**
Cardiogenic shock	89 (8%)	37 (12%)	52 (7%)	**0.009**
In-hospital death	30 (3%)	16 (5%)	14 (2%)	**0.004**
In-hospital treatment				
Invasive ventilation (960/1,071)^a^	141 (15%)	75 (27%)	66 (10%)	** < 0.001**
IABP (1,056/1,071)^a^	15 (1.4%)	5 (1.6%)	10 (1.3%)	0.778
Lenght of stay	7 (5, 10)	8 (5, 11)	6 (5, 9)	** < 0.001**
**All-cause mortality**				
Follow up (days)	576 (27,1668)	486 (14,1607)	605 (36,1733)	0.193
Long-term	155 (14%)	69 (22%)	86 (11%)	** < 0.001**

Data are presented as no. (%), median (interquartile range).

Bold values are statistically significant.

*IABP* intra-aortic balloon pump.

^a^Number of patients with available data.

**Table 4:** Univariate and multivariable logistic regression analysis for prediction of in-hospital complications.

Variable	Univariate		Multivariable	
	OR (95% CI)	p	OR (95% CI)	p
Age (per 10 year)	1.17 (1.01–1.35)	** < 0.034**	NS	NS
Male	2.74 (1.74–4.31)	** < 0.001**	2.9 (1.65–5.1)	** < 0.001**
Dyspnea	3.51 (2.49–4.92)	** < 0.001**	3 (1.96–4.62)	** < 0.001**
Physical trigger	1.23 (0.87–1.74)	0.235	–	–
History of cancer	1.07 (0.66–1.74)	0.770	–	–
Diabetes	2.01 (1.37–2.93)	** < 0.001**	NS	NS
Pulmonary disease	2.44 (1.66–3.59)	** < 0.001**	1.75 (1.1–2.8)	**0.02**
EF (per 10% decrease)	2.21 (1.8 2.71)	** < 0.001**	2.13 (1.66–2.74)	** < 0.001**
Atrial fibrillation	2.96 (1.89–4.63)	** < 0.001**	2.27 (1.31–3.93)	**0.004**
Apical ballooning	1.31 (0.77–2.21)	0.317	-	-

Bold values are statistically significant.

*OR* odds ratio, *CI* confidence interval, *EF* left ventricular ejection fraction, *NS* non-significant.

**Figure 1:**
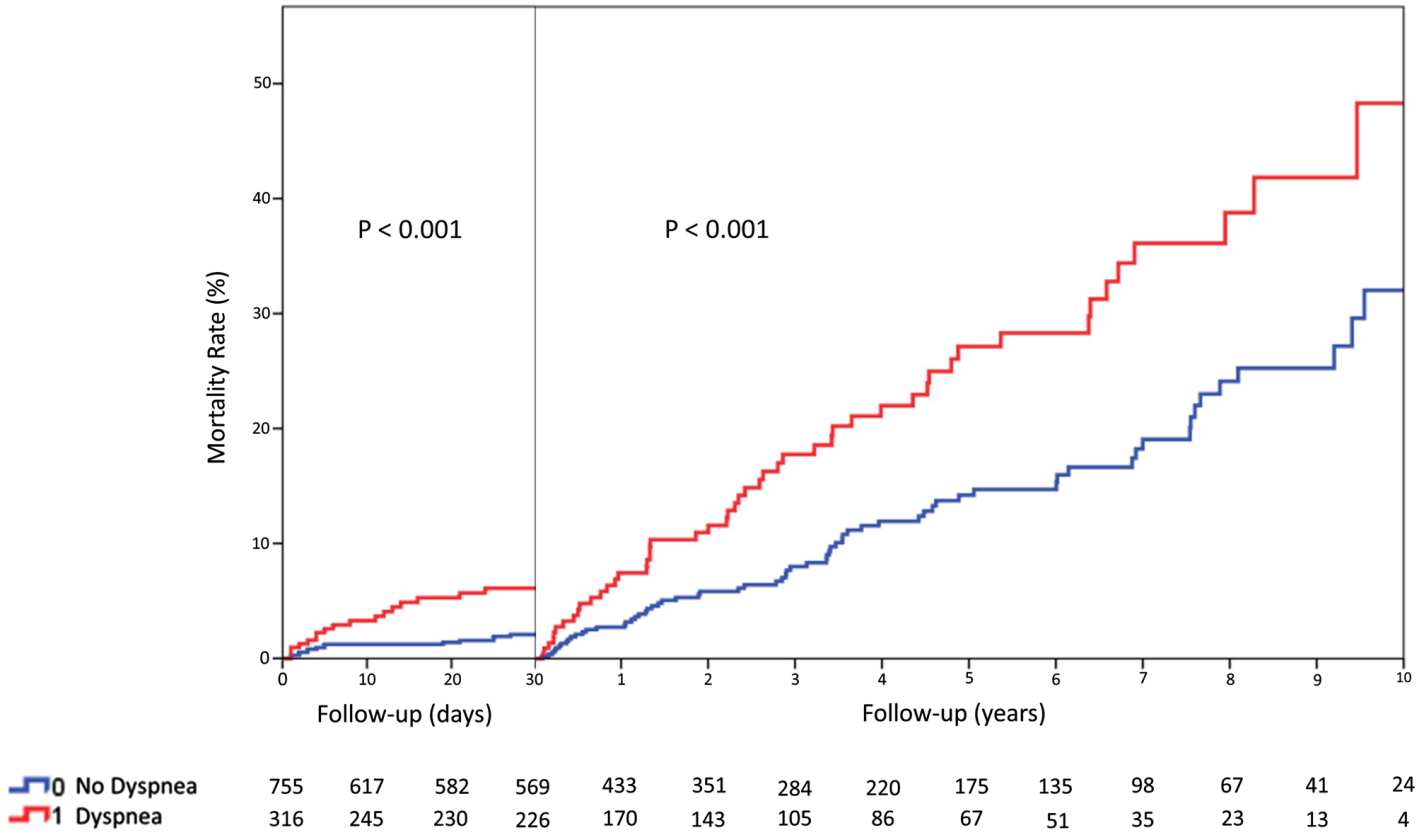
Kaplan–Meier curves showing survival rate at 30 days and long-term follow-up among patients admitted with takotsubo syndrome with or without dyspnea.

During a median follow up of 576 days (interquartile range: 27,2), TTS patients with dyspnea at admission had significantly higher long-term mortality rates compared with those without (22% vs. 11%; p < 0.001) (Table 2). Landmark analysis at 30 days showed significant increase in mortality rate among patients presenting with dyspnea both in the acute phase and in the long term (p < 0.001 for both, Fig. 1). Kaplan–Meier plot illustrates mortality curves progressively diverging during the length of follow-up (Fig. 1). In multivariable Cox-regression analysis, dyspnea at admission was also a significant predictor of the secondary end-point long-term mortality [HR 2.02 (95% CI 1.37–2.98); p < 0.001] (Table 5). In addition, age, male sex, the presence of malignancies or pulmonary diseases, lower LVEF and the occurrence of cardiogenic shock during the acute phase were identified as secondary end-point determinants.

**Table 5:** Univariate and multivariable Cox-regression analysis for predictors of long-term mortality.

Variable	Univariate		Multivariable	
	HR (95% CI)	p	HR (95% CI)	p
Age (per 10 year)	1.75 (1.47–2.01)	** < 0.001**	1.57 (1.28–1.91)	** < 0.001**
Male	2.61 (1.69–4)	** < 0.001**	1.89 (1.13–3.18)	**0.015**
Dyspnea	2.19 (1.58–3.02)	** < 0.001**	2.02 (1.37–2.98)	** < 0.001**
Physical trigger	1.35 (0.97–1.89)	0.078	NS	NS
History of cancer	2.65 (1.82–3.89)	** < 0.001**	2.91 (1.8–4.72)	** < 0.001**
Diabetes	1.68 (1.12–2.42)	**0.005**	NS	NS
Pulmonary disease	2.53 (1.62–3.41)	** < 0.001**	1.67 (1.1–2.51)	**0.014**
EF (per 10% decrease)	1.68 (1.4–2)	** < 0.001**	1.77 (1.43–2.2)	** < 0.001**
Atrial fibrillation	2.26 (1.51–3.39)	** < 0.001**	NS	NS
Apical ballooning	1.9 (1.11–3.24)	**0.018**	NS	NS
Cardiogenic shock	4.65 (3.22–6.72)	** < 0.001**	4.1 (2.64–6.39)	** < 0.001**

Bold values are statistically significant.

*HR* hazard ratio, *CI* confidence interval, *EF* left ventricular ejection fraction, *NS* non-significant.

## Discussion

We report incidence of dyspnea and prognostic implication among a multicenter international registry of patients admitted with TTS. The main results of the present study are as follows: (I) dyspnea at hospital admission is present in one third of patients admitted with TTS; (II) it is associated with coexisting comorbidities and worse cardiac function during the acute phase; and (III) dyspnea is an independent predictor of in-hospital complications and long-term mortality in TTS patients.

### Incidence and determinants of dyspnea

In our analysis, approximately one third of TTS patients had dyspnea at admission, a result in keeping with previous TTS studies^17^ and comparable to what observed among ACS patients^13^. Several cardiac mechanisms could be responsible for dyspnea in the setting of an acute heart failure syndrome^18^. In TTS, these include systolic and diastolic dysfunction^19^, mitral insufficiency due to papillary muscles dysfunction^20^, atrial fibrillation^21^, and heart rate^9^. Accordingly, in our population we found that lower LVEF was independently associated with dyspnea. On the other hand, the symptom dyspnea not only represents underlying cardiovascular dysfunction, but it could reflect more variegate interactions, being influenced by the presence of coexisting comorbidities^22^ which are quite common in patients with TTS^17^. Consistently, we found TTS patients with dyspnea to be older and to have higher prevalence of physical triggers (i.e. underlying illness as a cause of the acute TTS episode), which may be directly linked to the symptom breathlessness^22^. Moreover, pulmonary diseases and diabetes were independently associated with the occurrence of dyspnea, with the latter possibly contributing through myocardial fibrosis, dysfunctional remodeling and associated diastolic dysfunction^23^, leading to augmented LV filling pressures and pulmonary fluid accumulation as previously reported in the setting of TTS^14^.

### Prognostic relevance of dyspnea

In the present study, patients with dyspnea at admission had increased rates of in-hospital complications. Though dyspnea is intuitively linked to some of the components of the composite primary end-point that could have been already present at the time of symptom assessment, it is essential to highlight that complications could also develop later during hospitalization^24^. Therefore, earlier risk stratification might identify high-risk individuals that could most benefit from a more intensive and prolonged management^11^.

Moreover, long-term mortality rate was higher in patients exhibiting dyspnea at presentation, even after excluding those events during the first month, with mortality curves progressively diverging during follow-up. These findings indicate that TTS patients admitted with symptoms of decompensated heart failure suffer a worse prognosis even after LV systolic function is expected to have largely recovered, in keeping with a significant body of evidence suggesting that in TTS the severity of cardiac involvement correlates with worse prognosis in the long-term^24–30^. One potential explanation is that dyspnea at presentation, being associated to higher degree of cardiac dysfunction, represents a marker of more severe TTS episodes that can lead to long-term sequelae and subsequent heart failure phenotype^31^. Indeed, impaired cardiac mechanics^32^, diffuse fibrosis^31^ and inflammatory activation^33^ have been described after the acute phase, suggesting that a subgroup of patients might be characterized by incomplete recovery. Additionally, it has been suggested that TTS individuals prone to develop hemodynamic instability in the acute phase may be characterized by a pre-existing concealed decreased cardiac reserve^24^, possibly influencing long-term outcome^34^. Hence we hypothesize that, in the vulnerable subset of patients with dyspnea at presentation, comorbidities may act in a synergistic fashion with TTS functional abnormalities, resulting in greater cardiac dysfunction and decompensation during the acute phase, and worse prognosis in the long-term. Accordingly, the prognostic relevance of comorbidities is supported by results of both our and other studies^14,35–37^, and further corroborated by the fact that long-term mortality in TTS is mainly non-cardiovascular^6,10,24,29^.

In conclusion, dyspnea at presentation, reflecting both the TTS patient’s cardiac function and comorbidity burden, might potentially be a simple and easily evaluable parameter to identify those individuals with a worse prognosis.

## Conclusions

The occurrence of dyspnea at hospital admission is associated with higher degree of cardiac dysfunction and comorbidity burden. Furthermore, presence of dyspnea is associated with complicated in-hospital course and worse long-term prognosis, potentially making symptom assessment an easy and valuable tool in the search of reliable risk-stratification models for TTS patients.

## Limitations

Though the present observational study included one of the largest cohort of TTS patients to date, the generalizability might be limited, and our results should be considered hypothesis generating to be confirmed by further and targeted studies. Self-reported symptoms assessment is a mere qualitative measure of patient’s decompensation, nonetheless this is a key parameter to investigate a suspected heart failure condition and is commonly used in daily clinical practice. Though dyspnea and chest pain are by far the commonest symptoms in TTS, we could not provide data on other affection, such as neurovegetative symptoms, in the whole of our cohort. We do not have specific data on time to onset of in-hospital complications, hence we cannot exclude their presence at the same time as the dyspnea variable was collected; Due to the large number of centers involved in the registry, with a quite heterogeneous approach to blood samples analysis, we could not provide a detailed analysis of biomarkers profile, including inflammatory markers, in the whole of our cohort.

## Supplementary information

Supplementary Information
