# Supplementary material for: Incidence, determinants and prognostic relevance of dyspnea at admission in patients with Takotsubo syndrome: results from the international multicenter GEIST registry
Source: Sci Rep. 2020 Aug 12;10:13603. doi: 10.1038/s41598-020-70445-9 (PMC7424520; doi:10.1038/s41598-020-70445-9)
Supplement: Supplementary file 1 — Supplementary Information [file 41598_2020_70445_MOESM1_ESM.docx]

**Supplementary Table 1:** prevalence of dyspnea within specific physical trigger subgroups as describes by Uribarri et al.^1^. Details for the specific kind of stress were available in 331 out of 351 patients with physical trigger.

| Physical Trigger subgroup | Overall (n=331) | Dyspnea (n=142) | No Dyspnea (n=189) | P |
| --- | --- | --- | --- | --- |
| Infectious | 71 (21%) | 31 (22%) | 40 (21%) | NS |
| Neurological disorders | 16 (5%) | 3 (2%) | 13 (7%) | **<0.05** |
| Surgical/Intervention | 58 (17%) | 21 (15%) | 37 (20%) | NS |
| Physical activity/Trauma | 139 (42%) | 52 (47%) | 87 (46%) | NS |
| Hypoxia | 57 (14%) | 35 (25%) | 12 (6%) | **<0.05** |

Not significant (NS)

**Supplementary Reference**

1. Uribarri A, Núñez-Gil IJ, Conty DA, Vedia O, Almendro-Delia M, Duran-Cambra A et al. Short- and Long-Term Prognosis of Patients With Takotsubo Syndrome Based on Different Triggers: Importance of the Physical Nature. J Am Heart Assoc. 2019;8:e013701.
